# Street adolescents in low income setting exposed to hepatitis B and C, and disadvantaged by lifestyle: a Togolese cross-sectional study

**DOI:** 10.1186/s12889-024-19415-8

**Published:** 2024-07-16

**Authors:** Arnold Junior Sadio, Valentine Marie Ferré, Oumarou I. Wone Adama, Harold Régis Kouanfack, Anoumou Claver Dagnra, Amivi P. Amenyah-Ehlan, Laté Mawuli Lawson-Ananissoh, Diane Descamps, Charlotte Charpentier, Didier Koumavi Ekouevi

**Affiliations:** 1 Faculty of Health Sciences, Department of Public Health, University of Lomé, Center for Training and Research in Public Health, Lomé, Togo; 2 African Center for Research in Epidemiology and Public Health (CARESP), Lomé, Togo; 3 Global Health in the Global South (Inserm UMR 1219, IRD EMR 271), Bordeaux Population Health, Université de Bordeaux, Bordeaux, France; 4 Service de Virologie, Université Paris Cité, INSERM, IAME, UMR 1137, AP-HP, Hôpital Bichat- Claude Bernard, Paris, F-75018 France; 5 Faculty of Health Sciences, Department of Fundamental Sciences, University of Lomé, Lomé, Togo; 6 Laboratory of Molecular Biology and Immunology (BIOLIM), University of Lomé, Lomé, Togo; 7 Department of Medicine and Medical Specialties, University of Lomé, Lomé, Togo; 8 Hepatology and Gastroenterology Unit of the Campus Teaching Hospital of Lomé, Lomé, Togo

**Keywords:** Lifestyle, Hepatitis B and C viruses, Immunization profile, Street adolescents, Togo

## Abstract

**Background:**

In Togo, few data are available on viral hepatitis in street adolescents, a vulnerable population due to their lifestyle. The aim of this study was to describe the lifestyle of street adolescents (sexual practices and drug use), to estimate the prevalence of hepatitis B and C viruses, and to describe their HBV immunization profile in Togo.

**Methods:**

A cross-sectional study was conducted in Lomé (Togo) in July 2021. Street adolescents aged between 13 and 19 years were included. A questionnaire was used to document lifestyle. ELISA tests were performed for Hepatitis B surface antigen (HBsAg), Hepatitis B core and surface antibodies (anti-HBc, anti-HBs), and antibodies against hepatitis C virus (anti-HCV).

**Results:**

A total of 299 adolescents (5.4% female) with a median age of 15 years (IQR: 14–17) were included. Of these, 70.6% (211/299) were sexually active and 70.6% (149/211) had not used a condom during their last sexual intercourse. Drug use was reported by 42.1% of the adolescents. The most used substances were cannabis (39.0%), cocaine (36.6%), glue solvents (19.5%), and tramadol (11.4%). However, cocaine use may have been overestimated due to information bias. Current HBV infection (HBsAg+) was detected in 3.7% (95%CI: 1.9–6.5) of the adolescents. Isolated anti-HBc + was present in 5.3%. All three HBV markers (HBsAg, anti-HBs, and anti-HBc) were negative in 71.6% of adolescents. Anti-HCV was detected in 4.7% of adolescents.

**Conclusion:**

Nearly one in 10 street adolescents has markers for HBV contact/current infection, and approximately 72% of street adolescents may still be infected with HBV, as they have no HBV markers. HCV is also circulating in this population. Given the reported high-risk sexual practices and high levels of drug use, there is an urgent need to develop integrated strategies to prevent infections, including HBV, and drug dependence in this population.

## Background

Viral hepatitis are a public health concern and the seventh leading cause of death worldwide [1]. According to the World Health Organization (WHO), they are responsible for around 1.4 million deaths every year [1]. Hepatitis B and C viruses cause chronic disease in hundreds of millions of people and together are the leading cause of liver cirrhosis, cancer and death from viral hepatitis [2–4]. In 2019, the number of people chronically infected with the hepatitis B virus (HBV) was estimated at 296 million, and those infected with the hepatitis C virus (HCV) at 58 million, including 3.2 million adolescents and children [2].

In 2020, the African region accounted for 26% of the global burden of disease due to hepatitis B and C, with 125,000 associated deaths. HCV, on the other hand, spares no region, with major differences between and within countries [1]. Vertical transmission has been identified as one of the causes of the high prevalence of hepatitis B infection in sub-Saharan Africa. Currently, 33 countries have a hepatitis B prevalence of over 1% in children under 5 years of age, representing a slight improvement on the 40 countries surveyed in 2019 [5]. Nevertheless, a safe and effective vaccine is available, providing 98–100% protection against the disease [5]. In the African region, vaccination coverage of children against hepatitis B virus is currently estimated at 72%, well below the global target of 90%, at which level the virus will no longer pose a threat to public health [5].

In sub-Saharan Africa, there are very few data on the prevalence of viral hepatitis in children and adolescents. In settings where the prevalence of hepatitis B is high, mother-to-child transmission of hepatitis B is likely to be the main mode of transmission [6]. However, a high level of transmission can also be observed in childhood among people who have not been vaccinated. Interventions aimed at reducing the incidence and prevalence of viral hepatitis must be initiated as early as possible in childhood or adolescence among non-immune individuals. Unfortunately, because of their lifestyle and limited access to health services, street adolescents do not have the opportunity to benefit from such interventions. Street adolescents are clearly distinguished from other adolescents by the fact that they are the most exposed to the risk of sexually transmitted infections (STIs), including HIV and viral hepatitis, whether through their sexual behavior or drugs use [7]. In fact, street adolescents often engage in risky sexual behavior, including early sexual activity, infrequent condom use, multiple partners and sex for survival (sex in exchange for money, food, shelter or protection) [8–10]. Regarding drugs, the prevalence of drugs use by adolescents in sub-Saharan Africa was estimated at 41.6%. Among the regions most affected were Central Africa (55.5%) and West Africa (38.3%) [11].

While data on sexuality and drugs use among adolescents in the general population seem to be available and easily accessible, few data exist on street adolescents, in West Africa. The aim of this study was to describe the lifestyle, including sexual practices and drugs use, of street adolescents, estimate the prevalence of hepatitis B and C viruses, and describe their HBV vaccination profile in Togo.

## Methods

### Study design and period

This was a cross-sectional study carried out in July 2021 in Lomé, the capital city of Togo. This city alone accounts for 51.4% of the street adolescents in Togo [12].

### Study population

This was an ancillary study, based on the STADOS project, whose main objective was to assess the acceptability and feasibility of HIV self-testing among street adolescents in Togo [13]. The target population was all adolescents (i) regardless of sex, (ii) who had been living on the street for at least three months, (iii) aged between 13 and 19 and (iv) who had agreed to take part in the study.

### Data collection

No questionnaire was developed specifically for this ancillary study, and all socio-behavioural and sexual variables were collected using the questionnaire developed for the STADOS princeps study. Information on sexual practices, drug use and routes of drug consumption was obtained using a face-to-face questionnaire administrated by medical students trained before the survey. The questionnaire was produced in two versions, one French and one English. For those who only understood or spoke the vernacular (*Ewe*), questions were translated and administered in *Ewe*.

### Specimen collection and laboratory analysis

At the end of the questionnaire, a blood sample was collected in an EDTA tube (5 ml). Plasma samples obtained after centrifugation were aliquoted into cryotubes and stored at -70 °C. They were then sent to the virology laboratory at Hôpital Bichat-Claude Bernard (Paris, France) to test for HBV surface antigen (HBsAg), core antibodies (anti-HBc), surface antibodies (anti-HBs) and antibodies against hepatitis C virus using Anti-HBs, Anti-HBc II and HBsAg Qualitative II Reagent Kits for HBV and Anti-HCV Reagent Kit for HCV on an Alinity i automate (Abott ©, Germany).

### Operational definitions

The profile of adolescents with regard to hepatitis B virus was defined as follows [14–16]: (i) **Current HBV infection**: positive surface antigen HBs (HBsAg+), negative surface antibody (anti-HBs-) and positive core antibody (anti-HBc+); or isolated HBsAg+; (ii) **Resolved infection**: HBsAg-, anti-HBs+, anti-HBc+; (iii) **HBV-vaccinated profile**: HBsAg -, anti-HBc-, anti-HBs+; (iv) **Uninfected and unvaccinated profile**: HBsAg-, anti-HBs- and anti-HBc-.

A positive anti-HCV test was interpreted as a contact marker, indicating that the adolescent had been in contact with HCV, without specifying how long the infection had lasted.

### Statistical data analysis

Descriptive statistics were carried out, and the results presented in tables of numbers and proportions for categorical variables. Quantitative variables were presented in median form with their interquartile range (IQR). Categorical variables were compared using the chi-square and/or Fisher test, and the Wilcoxon test was used to compare quantitative variables. All analyses were performed using R version 4.2.1 statistical software.

### Ethical considerations

This study was approved by Togo’s National Program for the Fight against AIDS, Viral Hepatitis and STIs (PNLS/HV/IST) and Togo’s Bioethics Committee for Health Research (N°012/2020/CBRS).

Participants over 18 years of age provided consent. For minors (< 18 years), in addition to their imperatively required assent, persons in charge of adolescents (in this case non-governmental organizations) were asked to sign consent to participate in the study. This approach was recommended by the WHO in its guidelines on ethical aspects to consider when planning and reviewing research on adolescent sexual and reproductive health [17]. All the adolescents benefited from awareness-raising sessions and condom distribution at the inclusion site (NGO *Jade pour la vie*). Beyond the project’s scope, ‘Jade pour la vie’s adolescent and youth listening center remained open to participants, providing a safe and supportive space for them to seek guidance and assistance, including comprehensive sexual and reproductive health education and counseling.

## Results

### Socio-demographic characteristics and sexual practices of street adolescents

A total of 299 street adolescents, 5.4% of them female, were recruited in this study. The median age (IQR) was 15 years (14–17).

Of the 299 street adolescents interviewed, 211 (70.6%) reported having had sexual intercourse. The median age (IQR) at first intercourse was 13 years (13–15). Nearly a third of the street adolescents had accumulated more than 5 sexual partners since their first experience.

In addition, 85.7% said that their first experience was motivated by a personal desire, while 9.0% of adolescents would have been coerced. Sexual intercourse in exchange for services, or money was reported by 8.5% of the surveyed adolescents. Among the 211 sexually active adolescents, only 62 (29.4%) had used a condom during their last sexual intercourse. Table 1 presents the socio-demographic characteristics and sexual practices of the street adolescents included in this study.

**Table 1:** Socio-demographic characteristics and sexual behaviors of street adolescents, Togo, 2021

	*N*	Proportion (%)
**Age (Years)**		
Median [IQR]	15 [14.0–17.0]	
< 15	106	35.5
≥ 15	193	64.5
**Sex**		
Female	16	5.4
Male	283	94.6
**Education level**		
None	21	7.0
Primary	122	40.8
Secondary	122	40.8
University	34	11.4
**Nationality**		
Others^§^	53	17.7
Togolese	246	82.3
**Sexually active**		
No	88	29.4
Yes	211	70.6
**Age at first sexual intercourse (Years)**		
Median (IQR)	13 [13.0–15.0]	
**Number of partners since first sexual intercourse**		
< 2	109	54.2
3–4	36	17.9
≥ 5	56	27.9
**Circumstances of first sexual intercourse**		
I wanted the experience of sexual intercourse	180	85.7
I was coerced	19	9.0
For money or services	3	1.4
Other^$^	8	3.8
**Sexual intercourse in exchange for services or money since the first sexual experience**		
No	193	91.5
Yes	18	8.5
**Condom use during last sexual intercourse**		
No	149	70.6
Yes	62	29.4

^§^Nigerian, Ghanaian, Beninese

^$^Out of curiosity or to do what others do

### Alcohol and drug use among street adolescents

**Fig. 1:**
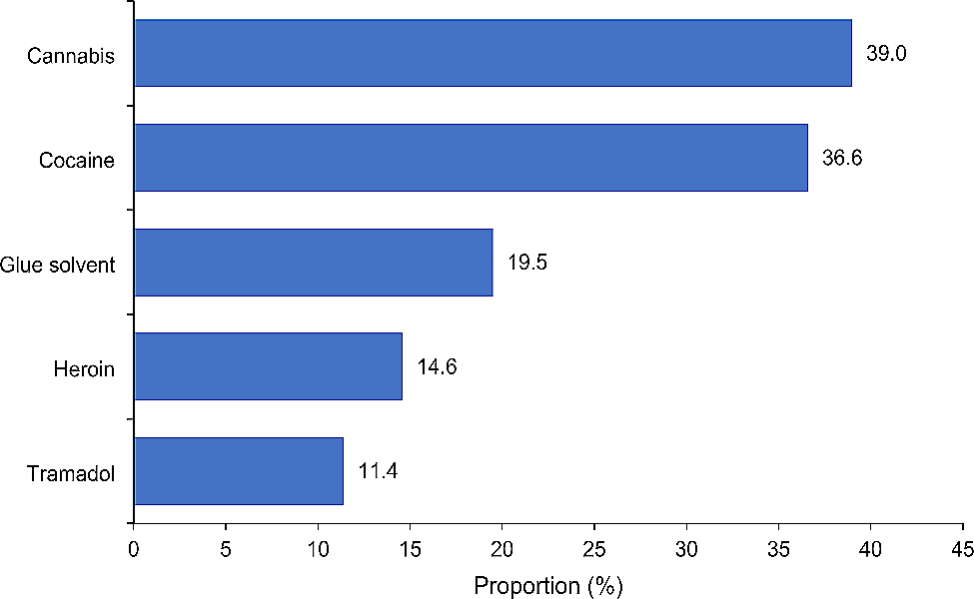
Types of drugs used by street adolescents, Togo, 2021

About their alcohol consumption, 25.9% of street adolescents reported drinking two or three times a week, and 3.7% every day. Almost half (42.1%) of street adolescents reported drugs use (Table 2). Cannabis, cocaine and glue solvents were the substances most frequently reported by street adolescents, in proportions of 39.0%, 36.6% and 19.5%, respectively (Fig. 1). One adolescent in ten (11.4%) reported having used tramadol.

**Table 2:** Use of psychoactive substances by street adolescents, Togo, 2021

	*N*	Proportion (%)
**Alcohol consumption**		
Do not wish to answer	2	0.7
No	83	27.8
Yes, rarely	145	48.5
Yes, often	69	23.1
**Frequency of alcohol consumption**		
Once a week	133	70.4
Two or three times a week	49	25.9
Every day	7	3.7
**Drug use**		
No	172	57.9
Yes	125	42.1
**Drug administration route**		
Injectable	6	4.8
Non injectable	118	95.2

### Viral hepatitis B among street adolescents

Current HBV infection (HBsAg+) was detected in 11/299 street adolescents (3.7%; 95%CI: 1.9–6.5), all males. For 16/299 adolescents (5.4%), isolated anti-HBc + was recorded. In all, almost one in 10 street adolescents (9,0%) had a current HBV infection or an isolated anti-HBc+. HBV immunity (anti-HBs+) was observed in 19.3% (*n* = 58) of adolescents, including 8.0% (*n* = 24) of vaccinated profiles. Table 3 shows the various HBV markers in street adolescents.

**Table 3:** Hepatitis B in street adolescents, Togo, 2021

	Current HBV infection, *n* (%)	Isolated anti-HBc, *n* (%)	Resolved infection, *n* (%)	HBV-vaccinated profile, *n* (%)	Uninfected and unvaccinated profile, *n* (%)
	11 (3.7)	16 (5.4)	34 (11.3)	24 (8.0)	214 (71.6)
**Age (Years)**					
Median [IQR]	15 [14.0–17.0]	17 [17.0–18.2]	17 [15.0–18.0]	15 [13.0–18.0]	15 [14.0–17.0]
< 15	2 (18.2)	3 (18.8)	7 (20.6)	10 (41.7)	84 (39.3)
≥ 15	9 (81.8)	13 (81.2)	27 (79.4)	14 (58.3)	130 (60.7)
**Sex**					
Female	0 (0.0)	1 (6.2)	1 (2.9)	1 (4.2)	13 (6.1)
Male	11 (100.0)	15 (93.8)	33 (97.1)	23 (95.8)	201 (93.9)
**Education level**					
None	2 (18.2)	2 (12.5)	3 (8.8)	2 (8.3)	12 (5.6)
Primary	8 (72.7)	8 (50.0)	12 (35.3)	11 (45.8)	83 (38.8)
Secondary	1 (9.1)	5 (31.2)	17 (50.0)	8 (33.3)	91 (42.5)
University	0 (0.0)	1 (6.2)	2 (5.9)	3 (12.5)	28 (13.1)
**Nationality**					
Others^§^	1 (9.1)	5 (31.2)	6 (17.6)	5 (20.8)	36 (16.8)
Togolese	10 (90.9)	11 (68.8)	28 (82.4)	19 (79.2)	178 (83.2)
**Sexually active**					
No	1 (9.1)	2 (12.5)	10 (29.4)	8 (33.3)	67 (31.3)
Yes	10 (90.9)	14 (87.5)	24 (70.6)	16 (66.7)	147 (68.7)
**Age at first sexual intercourse (Years)**					
Median [IQR]	13 [12.0–15.5]	13 [12.0–15.0]	13 [12.0–15.0]	13 [12.0–15.0]	13 [12.0–15.0]

**Current HBV infection**: positive surface antigen HBs (AgHBs+), negative surface antibody (anti-HBs-) and positive nucleocapsid antibody (anti-HBc+) or Isolated HBsAg+

**Isolated anti-HBc**: Isolated anti-HBc+

**Resolved Infection**: HBsAg-, anti-HBs+, anti-HBc+

**HBV-vaccinated profile**: HBsAg -, anti-HBc -, anti-HBs+

**Uninfected and unvaccinated profile**: HBsAg-, anti-HBs- and anti-HBc-

^$^Ghanaian, Nigerian, Beninese

### Viral hepatitis C among street adolescents

Regarding HCV, 14 street adolescents were seropositive, corresponding to a proportion of 4.7% (95%CI: 2.8–7.7) (Table 4). Ten were sexually active, with a median age (IQR) at first sexual intercourse of around 14 years (13–15).

**Table 4:** Hepatitis C in street adolescents, Togo, 2021

	Anti-HCV		*p*
	Negative, *n* (%)	Positive, *n* (%)	
	285 (95.3)	14 (4.7)	
**Age (Years)**			0.86
Median (IQR)	15 (14.0–17.0)	16 (13.5–17.8)	
			0.78
< 15	102 (35.8)	4 (28.6)	
≥ 15	183 (64.2)	10 (71.4)	
**Sex**			> 0.99
Female	16 (5.6)	0 (0.0)	
Male	269 (94.4)	14 (100.0)	
**Education level**			0.24
None	19 (6.7)	2 (14.3)	
Primary	114 (40.0)	8 (57.1)	
Secondary	119 (41.7)	3 (21.5)	
University	33 (11.6)	1 (7.1)	
**Nationality**			0.48
Others^$^	52 (18.2)	1 (7.1)	
Togolese	233 (81.8)	13 (92.9)	
**Sexually active**			> 0.99
No	84 (29.5)	4 (28.6)	
Yes	201 (70.5)	10 (71.4)	
**Age at first sexual intercourse (Years)**			0.10
Median [IQR]	13 [12.0–15.0]	14 [13.0–15.0]	

^$^Ghanaian, Nigerian, Beninese

## Discussion

This study to describe the prevalence of viral hepatitis B and C among street adolescents, also documented the lifestyle, i.e. sexual practices and drug use among these adolescents in Togo. This is a particularly hard-to-reach population for which very few data exist in West Africa. To our knowledge, this is the first study on hepatitis B and C in this population in West Africa.

In terms of sexual practices, almost 70% of street adolescents surveyed were sexually active, and of these, almost a third had accumulated more than 5 sexual partners since their first experience, while only 29.4% had used a condom at last sexual intercourse. Condom use among street adolescents in Togo is therefore relatively more inconsistent than that recorded in the population of adolescents aged 15 to 19 in sub-Saharan Africa [18]. In fact, according to a 2018 systematic review and meta-analysis of studies carried out in sub-Saharan Africa, the prevalence of condom non-use was estimated at 60% [18]. This low condom use, combined with multiple sexual partners, exposes street adolescents to poor sexual and reproductive health outcomes. These outcomes include unwanted pregnancies, abortions, and STIs, including HIV and viral hepatitis [19]. In addition to the non-use of condoms and the multiplicity of partners, other factors need to be taken into account when referred to sexual and reproductive health among adolescents, in particular drugs use [20].

The present study reported a significant pattern of cannabis, cocaine, glue solvents and tramadol use. While cannabis has been reported as the main substance used by street adolescents in other studies [21], this is not the case for cocaine. There are three possible explanations for such a high rate of cocaine used reported, which in fact is supposed to be an inaccessible substance for street adolescents: (i) the first element is due to the fact that street adolescents, through their activities, may be in contact with the adult mafia circles where these drugs are distributed. As a reminder, West Africa is now recognized as a hub for international cocaine traffic [22]; (ii) the second element is related to an information bias linked to the way the question on cocaine use was formulated. During the interviews, in order to document cocaine use, adolescents were asked whether they ever consumed or snorted “white powder”. In the street environment, this can lead to confusion, especially as tramadol, which is generally a white tablet, is sometimes crushed and snorted to obtain faster, more intense effects [23]. Although rare and poorly described in comparison with other substances, this type of consumption does occur with tramadol and can lead to even greater health risks for adolescents [23, 24]. Also, in sub-Saharan Africa and Togo in particular, tramadol, which is sold retail on the streets, is more accessible than other substances, which are generally more expensive [25]; (iii) A third element, also linked to information bias, is that the term “sniffed” used when documenting cocaine use led to confusion with sniffing glue solvents, and thus to over-reporting. In fact, like tramadol, glue solvents contained in glue are very easily accessible substances on the streets of sub-Saharan Africa. These last two hypotheses are in accordance with several studies that have reported a clear difference in consumption habits between street adolescents in low- and middle-income countries and street adolescents in developed countries. According to these studies, street adolescents in developed countries consume more hard and injectable drugs, which are not commonly used by those in low- and middle-income countries. [26–28].

In addition to the risk of STI’s linked to unsafe sexual relations facilitated by the drug consumption, other health risks are to be feared for these adolescents. In the case of tramadol, neurotoxic complications linked to oxidative stress have been described [29]. Elsewhere, glue solvents inhalation has been associated with an increased risk of sudden death and chronic visceral damage [30]. Inhalant abuse in general is also associated with desocialization in adolescents and young adults, which in the case of street adolescents could make social reintegration processes more difficult [30]. Interventions targeting this population in Togo must take these data into account in order to identify use and prevent complications linked to these drugs in this population.

This study also aimed to estimate the prevalence of viral hepatitis B and C among street adolescents in Togo. Overall, nearly one street teenager in 10 has been in contact with HBV, with 3.7% showing active infection. Although this prevalence is relatively high, it remains low compared with that observed in adult populations in Togo, where prevalence studies report estimates ranging from 15 to 35% [31, 32].

Also, immunity against HBV (anti-HBs Ab+) was observed in only 19.4% (*n* = 58) of adolescents, including 8.0% (*n* = 24) of vaccinated profiles. These results could be explained by the fact that the hepatitis B vaccine was introduced into Togo’s Expanded Program on Immunization in 2008, and to have benefited from such an intervention one would have had to have been born in 2008 or later, and therefore aged 13 or under at the time of the survey. However, we surveyed adolescents aged 13 to 19. Thus, the present study reports nearly 80% of street adolescents with no immunity to HBV. In view of the risky sexual behavior reported among this population in the present study, it is necessary, indeed urgent, to plan targeted HBV vaccination campaigns for this population. The data reported in this study, which is the only one in Togo to have estimated the prevalence of HBV markers among street adolescents, could serve as a basis for planning such an activity.

While it is possible to vaccinate to protect against HBV, this is not yet the case for HCV. Today, prevention against HCV is based on behavioral approaches, in particular a healthy lifestyle, better access to screening and improved quality of care for people at risk [33, 34]. Unfortunately, these approaches are difficult to apply to street adolescents, for whom a healthy lifestyle remains illusory and access to health services is limited. In the present study, 14 street adolescents had an HCV serology positive, corresponding to a prevalence of 4.7%. This observed prevalence is almost identical to that reported in Africa, where the prevalence of viral hepatitis C has been estimated at 5.3% [35]. This prevalence needs to be put into perspective, as although it may appear low, it is still higher than that reported in other WHO regions [35]. For a population such as street adolescents, who enter sexual life at an early age, have multiple partners, have low condom use and abuse various substances, there is a need to strengthen the interventions carried out by NGOs for these populations. This means going beyond HIV and integrating viral hepatitis prevention into the intervention package.

This study is one of the first to estimate the prevalence of viral hepatitis markers among street adolescents in sub-Saharan Africa, as well as their addictive and sexual behaviors. The results of this study tend to confirm that this is a population vulnerable to STIs, particularly viral hepatitis, due to their lifestyle. However, this study has some limitations, notably the absence of viral load estimates for viral hepatitis B and C to confirm possible viral replication for possible treatment eligibility. Also, the assessment of drugs use was probably subject to an information bias in the case of cocaine (considering the cost of this drug), which was probably confused with sniffing tramadol or sniffing solvents contained in glue. Another difficulty we encountered is related to the documentation of alcohol consumption, as the main existing tools are difficult to apply to street adolescents [36, 37].

## Conclusion

This study confirms that street adolescents in Togo are a vulnerable population due to their exposure to STI, including viral hepatitis, as well as their addictive and sexual behaviors. Nearly one in 10 street adolescents has markers for HBV contact/current infection. HCV also circulates in this population. Immunity against HBV is not optimal, and nearly seven in 10 of street adolescents have no immunity at all against HBV and need to be vaccinated against HBV considering the identified sexual risk behaviors. In addition, a high level of drugs use has been reported among them. Future interventions targeting this population must, in addition to integrating the prevention of viral hepatitis, identify the use and prevent the complications linked to drug abuse, in order to guarantee a future better social reintegration of these adolescents.

## Data Availability

The datasets used and/or analysed during the current study are available from the corresponding author upon reasonable request.
